# Laboratory profiles of patients hospitalized with COVID-19 pneumonia treated with tofacitinib or placebo: a post hoc analysis from the STOP-COVID trial

**DOI:** 10.31744/einstein_journal/2024AO0821

**Published:** 2024-10-17

**Authors:** Patrícia Oliveira Guimarães, Lucas Petri Damiani, Caio de Assis Moura Tavares, Ari Stiel Radu Halpern, J. Jasper Deuring, Luiz Vicente Rizzo, Otavio Berwanger

**Affiliations:** 1 Hospital Israelita Albert Einstein São Paulo SP Brazil Hospital Israelita Albert Einstein, São Paulo, SP, Brazil.; 2 Pfizer Inc. Rotterdam Netherlands Pfizer Inc., Rotterdam, The Netherlands.; 3 Imperial College London London United Kingdom Imperial College London, London, United Kingdom.; 4 George Institute for Global Health London United Kingdom George Institute for Global Health, London, United Kingdom.

**Keywords:** Tofacitinib, COVID-19, Coronavirus infections, Pneumonia, Lab results, Platelet count, Alanine transaminase, Aspartate aminotransferases, Janus kinase inhibitors

## Abstract

**Objective:**

Tofacitinib, an oral Janus kinase inhibitor, has been tested against a placebo in 289 patients with COVID-19 pneumonia. We analyzed the data from the tofacitinib- and placebo-treated patient cohorts to evaluate the laboratory profiles between baseline and day 7.

**Methods:**

We performed post hoc analyses on the following laboratory tests over time during the first 7 days after randomization: hemoglobin, leukocytes, neutrophils, lymphocytes, platelets, alanine aminotransferase, and aspartate aminotransferase.

**Results:**

Through the first 7 days after randomization, the levels of hemoglobin, white blood cells, neutrophils, and platelet counts were not significantly different between patients treated with tofacitinib or a placebo (all p>0.05). Non-significant differences were observed in aspartate aminotransferase levels over time between treatment groups, whereas alanine aminotransferase levels (U/L) were higher among tofacitinib-treated patients compared to placebo-treated patients (mean ratio, 1.30 [95% confidence interval (95%CI) = 1.14–1.48; p<0.01)].

**Conclusion:**

In patients with COVID-19 pneumonia, the use of tofacitinib compared to placebo did not result in clinically meaningful changes in blood counts or liver enzymes over the first 7 days after randomization.

**Registry of Clinical Trials:**

NCT04469114

## In Brief

In a post hoc analysis of the study of tofacitinib in hospitalized patients with COVID-19 pneumonia (STOP-COVID) trial, Guimaraes et al. evaluated the laboratory safety profile of tofacitinib use during the first 7 days of treatment in patients hospitalized with COVID-19 pneumonia compared with placebo. No clinically meaningful changes were observed in the value of white blood cells, lymphocytes, neutrophils, platelets, hemoglobin, or liver enzymes.

## Highlights

■Tofacitinib use did not result in meaningful changes in hematological parameters.

■Tofacitinib use did not lead to clinically meaningful changes in liver enzymes.

## Graphical abstract

**Figure:**
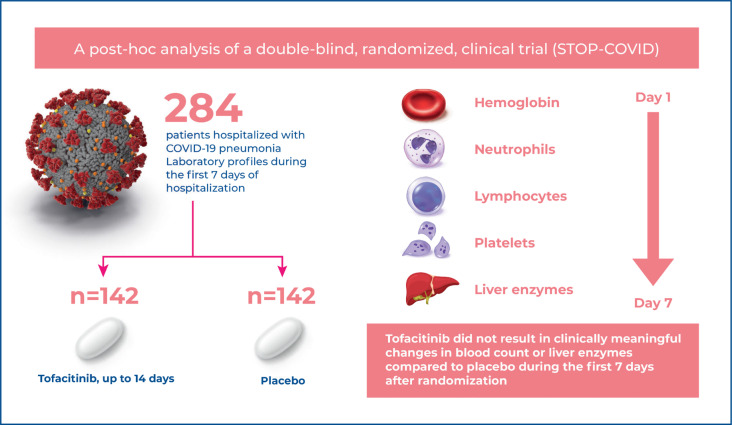

## INTRODUCTION

Tofacitinib is an oral Janus kinase (JAK) inhibitor that blocks intracellular cytokine pathways and is clinically indicated for the treatment of rheumatic diseases and ulcerative colitis.^(1)^ Due to its immunomodulatory properties, tofacitinib was tested against placebo in the study of tofacitinib in hospitalized patients with coronavirus disease 2019 (COVID-19) Pneumonia (STOP-COVID) trial, which included 289 patients hospitalized for COVID-19 pneumonia.^(2)^

Alterations in white blood cell counts, hemoglobin levels, and liver enzymes have been reported with tofacitinib use.^(3,4)^ Several laboratory abnormalities related to COVID-19 infection and associated with worse prognosis have been reported in this population.^(5)^

## OBJECTIVE

We analyzed data from the tofacitinib- and placebo-treated patient cohorts of the STOP-COVID trial to evaluate the laboratory profiles between baseline and day 7 after randomization.

## METHODS

STOP-COVID was a multicenter, randomized, double-blind, placebo-controlled trial that included patients with laboratory-confirmed severe acute respiratory syndrome coronavirus 2 (SARS-CoV-2) infection with radiographic evidence of COVID-19 pneumonia. The trial design and main results have been previously published.^(2)^ The study was approved by the research ethics committee of *Hospital Israelita Albert Einstein* (CAAE: 34810620.0.1001.0071; #4.147.123), and all participants provided written informed consent. Eligible patients were randomized to receive tofacitinib (10mg twice daily) or placebo for up to 14 days, or until hospital discharge. The dose reduction criteria have been previously described.^(2)^

We performed post hoc analyses on the following laboratory tests throughout the first 7 days after randomization: hemoglobin, leukocytes, neutrophils, lymphocytes, platelets, alanine aminotransferase (ALT), and aspartate aminotransferase (AST). Laboratory data are presented as mean and standard deviation. Mixed-effect models for repeated measures were used to estimate the mean difference in laboratory data (modeled from days 1 to 7 using a fourth-degree polynomial) and the interaction between time and treatment groups. All p values are shown for exploratory purposes, and p<0.05 was considered statistically significant.

## RESULTS

**Figure 1:**
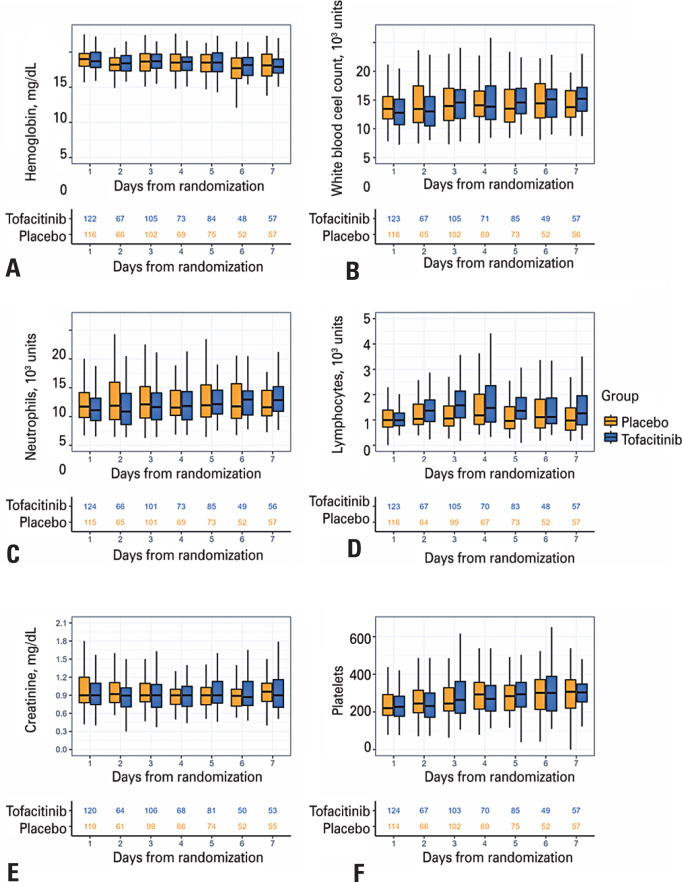
Daily values from baseline through day 7: A) hemoglobin (mg/dL), B) white blood cell count (10^3^ cells/mm^3^), C) neutrophils (10^3^ cells/mm^3^), D) lymphocytes (10^3^ cells/mm^3^), E) creatinine (mg/dL), and F) platelets (10^3^ cells/mm^3^). In each panel, the numbers in blue and yellow refer to the number of patients with results available for each day in the Tofacitinib and Placebo Groups, respectively

The present analysis included 284 patients who received the study drug (tofacitinib or placebo), with 142 patients in each treatment group. The median number of days of study drug exposure was 5 (interquartile range [IQR], 3–8 days) in the tofacitinib group and 6 (IQR, 4–10 days) in the placebo group. Throughout the first 7 days of hospitalization, hemoglobin, white blood cell, neutrophil, and platelet counts did not differ significantly between patients treated with tofacitinib and patients treated with placebo (all p>0.05; Figure 1). Lymphocyte counts, however, were slightly higher in patients treated with tofacitinib than in patients treated with placebo (mean difference, 0.30 [0.14–0.46]; p<0.01).

Non-significant differences were observed in AST levels (U/L) over time between the treatment groups (mean ratio [MR], 1.11 [95% confidence interval (95%CI) = 0.98–1.25]; p=0.11) whereas ALT levels (U/L) were significantly higher among patients treated with tofacitinib than those treated with placebo (MR, 1.30 [95%CI = 1.14–1.48]; p<0.01).

## DISCUSSION

In patients with COVID-19 pneumonia, the use of tofacitinib compared with placebo did not result in clinically meaningful changes in blood counts or liver enzyme levels over the first 7 days of randomization. Although elevated aminotransferase levels have been observed in several cohorts of COVID-19 patients, our results suggest that treatment with tofacitinib in this population does not promote a clinically relevant increase in liver enzymes. A significant limitation of this study is that no follow-up laboratory data were available after day 7. Previous modest changes in hematological parameters, however, have been reported in patients with rheumatic arthritis treated with tofacitinib for longer periods of time, which stabilized over time.^(3)^ Considering the physiological function of JAK proteins in hematopoiesis and the known association of the clinical course of COVID-19 with thrombocytopenia and lymphopenia, both of which are associated with poor outcomes in hospitalized COVID-19 patients,^(6)^ it is reassuring for clinicians to observe that treatment with tofacitinib did not appear to alter these hematological parameters. Examination of several laboratory parameters from randomization through treatment day 7 in the Tofacitinib and Placebo Groups contributed to the primary trial assessment and provided reassuring evidence regarding the safety profile of tofacitinib in patients hospitalized for COVID-19 pneumonia.

## CONCLUSION

The findings of this study suggest that despite a minimal mean increase in alanine aminotransferase levels over the first 7 days of treatment, no clinically relevant risk was noted of tofacitinib aggravating myelosuppression, liver injury, or both in patients hospitalized with COVID-19 pneumonia.

## References

[1] Xeljanz (tofacitinib) [package insert].

[2] Guimarães PO, Quirk D, Furtado RH, Maia LN, Saraiva JF, Antunes MO, Kalil R, Junior VM, Soeiro AM, Tognon AP, Veiga VC, Martins PA, Moia DD, Sampaio BS, Assis SRL, Soares RV, Piano LP, Castilho K, Momesso RG, Monfardini F, Guimarães HP, Ponce de Leon D, Dulcine M, Pinheiro MR, Gunay LM, Deuring JJ, Rizzo LV, Koncz T, Berwanger O, STOP-COVID Trial Investigators (2021). Tofacitinib in Patients Hospitalized with Covid-19 Pneumonia. N Engl J Med.

[3] Schulze-Koops H, Strand V, Nduaka C, DeMasi R, Wallenstein G, Kwok K (2017). Analysis of haematological changes in tofacitinib-treated patients with rheumatoid arthritis across phase 3 and long-term extension studies. Rheumatology (Oxford).

[4] Berhan A (2013). Efficacy, safety and tolerability of tofacitinib in patients with an inadequate response to disease modifying anti-rheumatic drugs: a meta-analysis of randomized double-blind controlled studies. BMC Musculoskelet Disord.

[5] Tong X, Cheng A, Yuan X, Zhong X, Wang H, Zhou W (2021). Characteristics of peripheral white blood cells in COVID-19 patients revealed by a retrospective cohort study. BMC Infect Dis.

[6] Richardson S, Hirsch JS, Narasimhan M, Crawford JM, McGinn T, Davidson KW, Barnaby DP, Becker LB, Chelico JD, Cohen SL, Cookingham J, Coppa K, Diefenbach MA, Dominello AJ, Duer-Hefele J, Falzon L, Gitlin J, Hajizadeh N, Harvin TG, Hirschwerk DA, Kim EJ, Kozel ZM, Marrast LM, Mogavero JN, Osorio GA, Qiu M, Zanos TP, the Northwell COVID-19 Research Consortium (2020). Presenting Characteristics, Comorbidities, and Outcomes Among 5700 Patients Hospitalized With COVID-19 in the New York City Area. JAMA.

